# Clinical Diagnosis, Treatment, and *ALDH7A1* Mutations in Pyridoxine-Dependent Epilepsy in Three Chinese Infants

**DOI:** 10.1371/journal.pone.0092803

**Published:** 2014-03-24

**Authors:** Zhixian Yang, Xiaoling Yang, Ye Wu, Jingmin Wang, Yuehua Zhang, Hui Xiong, Yuwu Jiang, Jiong Qin

**Affiliations:** Department of Pediatrics, Peking University First Hospital, Xicheng District, Beijing, China; Innsbruck Medical University, Austria

## Abstract

Pyridoxine-dependent epilepsy (PDE) is a rare autosomal recessive disorder that causes seizures in neonates and infants. Mutations of the *ALDH7A1* gene are now recognized as the molecular basis PDE and help to define this disease. Three Chinese children with PDE were clinically analyzed, followed by treatment and examination of the *ALDH7A1* mutations. The seizures of the 3 patients were all resistant to multiple anticonvulsants (2 to 7 types). For case 1, onset of seizures was at the age of 2 months. His seizures were well controlled by intravenous pyridoxine for several days at the age of 3 months 20 days and recurred at intervals of 13, 14 and 38 days after pyridoxine withdrawn for 3 times. At the age of 7 months, symptoms of PDE appeared and uninterrupted oral pyridoxine started. For case 2, her seizures occurred at 8 days after birth. After administration of multiple antiepileptic drugs observed ineffective, high-dose pyridoxine continuous therapy was taken at the age of 10 months and the significant treatment effect induced a diagnostic PDE. Seizure onset in case 3 was at the first day of birth. He experienced inadvertently pyridoxine therapy several times (first time at 2 days after birth) and achieved good therapeutic effect, which was confirmed by physicians until 4 months 10 days. The treatment process in our 3 patients suggested that pyridoxine should be early and purposefully used in patients with early onset seizures. *ALDH7A1* gene mutation analysis revealed compound heterozygous mutations in each case: heterozygous c.410G>A (p.G137E) and IVS11+1G>A in case 1, heterozygous c.952G>C (p.A318P) and heterozygous c.965C>T (p.A322V) in case 2, and heterozygous c.902A>T (p.N301I) and IVS11+1G>A in case 3. Only p.N301I was reported previously, all other mutations were novel. This is the first time to report cases of Chinese patients diagnosed with PDE by molecular genetic analysis.

## Introduction

Pyridoxine-dependent epilepsy (PDE, OMIM 266100) is a rare autosomal recessive disorder that was first described in 1954 by Hunt et al [1]. It is characterized by recurrent seizures mainly during neonatal or early infantile periods and is refractory to common anticonvulsants but responsive to high doses of pyridoxine. PDE was mapped on 5q31 in 2000 and in the same year, was shown to be accompanied by increased levels of pipecolic acid in plasma and cerebrospinal fluid [2], [3]. Early in 1994, the human gene *ALDH7A1*, which encodes antiquitin, was also mapped to 5q31 [4]. Although antiquitin was known to function as an aldehyde dehydrogenase, its physiological substrate was uncertain at that time. Based on the above studies and by analyzing 13 individuals with PDE, Mills et al [5] confirmed that PDE was caused by *ALDH7A1* gene mutations, and the concentration of alpha-aminoadipic semialdehyde was increased in cerebrospinal fluid, plasma or urine of PDE patients. *ALDH7A1* genetic defect results in alpha-amino adipic acid semialdehyde dehydrogenase enzyme (E.C. 1.2.1.31) deficiency in the lysine catabolic pathway, which further leads to the accumulation of alpha-aminoadipic semialdehyde, piperideine-6-carboxylate and pipecolic acids. By modification via an aldol condensation reaction, piperideine-6-carboxylate inactivates pyridoxal phosphate, the active form of pyridoxine, and this is necessary for the action of glutamic acid decarboxylase to synthesize the inhibitory neurotransmitter, γ-aminobutyrate. Pyridoxal phosphate is not only required for GABA decarboxylase enzyme function, but also 140 more enzymes. Seizures are likely caused by decreased function of GABA decarboxylase enzyme due to cofactor deficiency. Therefore, PDE patients need a large daily intake of pyridoxine to prevent seizures.

Based on the feasibility of biochemical tests and mutation analysis for PDE, numerous cases with classical or atypical presentation of PDE have been confirmed genetically in several countries. More than 60 different mutations within the 18 exons of the *ALDH7A1* gene have been published [5]–[12]. In this report, we describe 3 Chinese patients in whom genetic analysis revealed four novel mutations and one known mutation in the *ALDH7A1* gene.

## Materials and Methods

### Ethics Statement

This study was approved by Institutional Review Boards at Peking University First Hospital. Written informed consent was obtained from all participants or their parents in case of minors. All data were analyzed anonymously.

### Patients

Patient 1 was a male infant who was full-term at birth. The mother had hyperthyroidism during pregnancy. Apgar scores were 6, 7, and 10 after 1, 5, and 10 minutes. The newborn cried slightly, had hypotonia and was diagnosed with mild anoxic-ischemic encephalopathy and treated for several days. At 2 months old, recurrent seizures occurred mainly during sleep. There was unilateral deviation (left or right) of the eyes during the seizures, and they were often accompanied by unilateral or bilateral limb clonus. Each seizure lasted 1 to 2 minutes, and recurred 3 to 4 times each day. During this period, decreased thyroid stimulating hormone (0.23 IU/L, normal range: 0.34–5.06 IU/L) was observed, and secondary hypothyroidism was diagnosed. The patient was given sodium levothyroxine tablets (33 μg/day), but the seizures continued. The results of amino acid and organic acid analyses and brain MRI were all normal. At 8 days after seizure onset (at the age of 2 months 8 days), phenobarbital was administered (20 mg/kg intravenously followed by 5 mg/kg/day orally), and the seizure frequency decreased to 2 to 3 times over a period of 2 weeks. Vaproic acid was added for one week and then terminated due to therapeutic inefficiency and acute liver dysfunction.

Levetiracetam (165 mg/day) was used instead of phenobarbital and vaproic acid at the age of 3 months and his liver function improved gradually, but the seizures could not be controlled. The 4 hours EEG recording including awake and sleep states showed normal background activity, no interictal discharges and no seizures recorded. The use of pyridoxine (100 mg/day intravenous for one time) for 7 days was successful in controlling the seizures at the age of 3 months 20 days. Because the beneficial effect of pyridoxine could not be clearly established due to the administration of multiple antiepileptic drugs, the patient continued to have levetiracetam (375 mg/day) without oral pyridoxine. After 13 days, his seizures recurred. Clonazepam (0.25 mg/day) together with levetiracetam and pyridoxine (200 mg/day intravenous for 7 days) were effective in suppressing the seizures again. During the 2-week interval after withdrawal of pyridoxine, seizures recurred. At this time, intravenous pyridoxine (100 mg/day for 3 days) without adjusting the other antiepileptic drugs resulted in complete seizure control. Again, oral pyridoxine was still not administered and seizures recurred after 38 days. However, the seizures were controlled by intravenous pyridoxine (100 mg/day for 12 days) and increase of oral clonazepam (0.75 mg/day) together with levetiracetam (500 mg/day). At the age of 7 months, continuous oral pyroxidine therapy was initiated at a dose of 60 mg/day and seizures were controlled since then. At the age of 8 months, sodium levothyroxine was withdrawn because of normal level of thyroid stimulating hormone. After seizure free for 5 months and also based on the genetics diagnosis, clonazepam and levetiracetam were gradually withdrawn. Follow up to one year and 5 months, dose of pyroxidin increased to 120 mg/day without seizure relapse. The psychomotor development of this patient is in progress, but is still delayed for his age. He could pronounce “mother and father” subconsciously and could walk only under help.

Patient 2 was a female infant who was full-term at birth. Two degrees of the amniotic fluid pollution was found at the time of birth. Frequency seizures occurred at 8 days manifested limbs shake, followed by eyes slanting to the left or gaze fixed, purple lips, spittle, screaming. Sometimes one seizure episode lasted more than half an hour, or frequent seizures occurred within a few hours. Diazepam and phenobarbital showed only short-term effectiveness. At the 3 months after birth, valproic acid treatment started, and then topiramate, levetiracetam, clonazepam, carbamazepine in turn. All antiepileptic drugs showed no significant effect. During the course of the disease (at the age of about 4 to 6 months), oral pyridoxine (30 mg/day) was given intermittently, but the definite treatment time and the relationship with the seizure frequency were not clear. At the age of 10 months, together with topiramate, levetiracetam, clonazepam, oral pyridoxine (90 mg/day) were given and seizures could be controlled after a few days. Then, the diagnosis of PDE was suspected and confirmed by *ALDH7A1* genetic testing. All the antiepileptic drugs were gradually withdrawn and only oral pyridoxine (120 mg/day) maintained with seizure free since then.

It was interesting to notice that this patient's mother also suffered from hyperthyroidism, which was well controlled by taking propylthiouracil tablets during pregnancy. The patient had normal thyroid function after birth. Brain MRI showed white matter volume less than normal, frontal-parietal sulci and subarachnoid widened. Several EEG examinations all showed a small amount of multifocal discharges before high doses of pyridoxine treatment and then normal after treatment.

This patient has been followed up for 2 years (age of 2 years and 10 months). Her psychomotor development still lags behind normal children. She could walk a few steps alone, say very simple words, and identify simple objects.

Patient 3 was a boy. Threatened absorption was recorded at 6 months and 9 months pregnancy because his mother suffered from pregnancy-induced hypertension. His 2 years old sisters developed normally. His seizures manifested limbs shaking and eyes oblique side (left or right) occurred at 5 hours after birth. The seizures were controlled by intravenous pyridoxine and phenobarbital at 2 days after birth. The patient was then given intravenous ganglioside treatment for 3 courses (each course of 10 days, 10 days or 20 days interval). The purpose for using ganglioside was to “improve brain damage”. Seizures were controlled during this period. However, seizures recurred at 8 days after the third course of ganglioside treatment and were then controlled again at the first day by infusion therapy of ganglioside maintained for 14 days. After 20 days, seizures relapsed again. This time valproic acid treatment started and then add-on topiramate without any effect for the frequency seizures (several times per day). At the age of 4 months 10 days, by revisiting the treatment history, the astonishing thing was that after each infusion ganglioside, a group of liquids containing pyridoxine was used to flush the infusion tube in order to avoid the waste of ganglioside. Taking into account the control of seizures might due to pyridoxine instead of ganglioside, oral pyridoxine 120 mg/day was given and seizures were controlled in the day. The diagnosis of PDE was then confirmed by *ALDH7A1* genetic testing. Antiepileptic drugs withdrawal while maintaining oral pyridoxine (120 mg/day) led to seizure free since then.

Brain MRI showed delayed myelination and right ventricle slightly widened at the age of 4 months. Several EEG examinations showed a small amount of multifocal discharges before continuously high doses of pyridoxine treatment and then normal doses after continuous treatment.

At the age of 4 months, the patient showed retardation, such as no looking at objects and light, no head control. Follow-up one month after continuously high doses of pyridoxine treatment, the patient's mental state improve. He could look at objects and could laugh.

### Mutation Analysis of *ALDH7A1* gene

Genomic DNA was extracted from leukocytes of the 3 patients and from their parents using the standard methods, as described by Miller et al [13]. Each exon and exon-intron boundary of the *ALDH7A1* gene was amplified by polymerase chain reaction (PCR) using oligonucleotide primers.We designed these primers from the *ALDH7A1* genomic sequence (NM_001182.4) using Primer Premier 5.0 software (Premier Biosoft International, Palo Alto, CA) (Table 1). The PCR conditions were as follows: 100 ng of genomic DNA was amplified in a total volume of 50 μL containing 1 μL dNTP (10 mmol/L), 1 μL of each primer (10 μmol/L), 5 μL 10×PCR buffer (Takara, Dalian, China), and 1 μL of *Taq* DNA polymerase (2 U/μL, Tiangen, Beijing, China). The regular PCR cycling conditions were as follows: 94°C for 5 minutes, then 34 cycles at 94°C for 1 minute, 58°C for 15 seconds, and 1 minute at 72°C. The PCR reaction products were purified and sequenced using either sense or antisense primer by the BigDye Terminator cycle sequencing kit in the ABI PRISM 3730 genetic analyzer (PE Applied Biosystems, Foster City, CA).

**Table 1:** Primers required for the amplification of the human *ALDH7A1* gene.

*ALDH7A1*	Primers(5′---3′)	Product size(bp)
Exon 1F	CTCTATTTCAGCAGCTCTCAGG	327
Exon 1R	GGGGAGTCGGTAGGTCAGT	
Exon 2F	CCCGTTTGGTCTATTCCCTT	385
Exon 2R	TGCCTAATCTTTCTACGCTTCAC	
Exon 3F	GATTTCCGAAGTCTGGGTTG	458
Exon 3R	TGACCTGCTTATCAGATGTTTATG	
Exon 4F	TCTTCTCCTCTGTGCCTTCC	348
Exon 4R	GACAGCCTTATTGTCCACTCAA	
Exon 5F	GTGTGCCCGGTTGTACTTTAT	593
Exon 5R	TGTCTTACCCGTGGACTTGC	
Exon 6F	ATGAAGATCAGAGGCATAATGAA	389
Exon 6R	CTACTAAATAGGAAAGTGAGCAGGT	
Exon 7F	TGGCTGAGGTTAATGTAGTGATT	527
Exon 7R	CTGGCATGTTTCCAAAGAGTT	
Exon 8F	ATATGTGAGGAGAAATCCAGGTC	489
Exon 8R	AAATAAGAACCCAACAAAGTCCA	
Exon 9F	ACTTTCTTCCCTAACAACTTCCTC	472
Exon 9R	AATAAACATGACCACTGCCTCC	
Exon 10F	GGATAGGGTGTAATCAGGATAGG	464
Exon 10R	CAATGGCAATGGAAGTCTCA	
Exon 11F	ATCCTGGGTGACAAGAACAAA	433
Exon 11R	AAGGCAAAGTCCCTGAATGA	
Exon 12F	GACTTAGGGTTACAGTATGGGACA	875
Exon 12R	TAGGAGCAGACACGATACACCA	
Exon 13F	TCTTTGTTGCTGGGTCACTG	729
Exon 13R	GATGTCAAATGGGGTGGC	
Exon 14F	TGACTCCTTATTTCCATTTTTCTC	402
Exon 14R	ATCCACCATCATTTTCCTCTTAT	
Exon 15F	GCTTGAATGAAAGGAAGAATGTG	594
Exon 15R	AATCTCAGCCCCACTTGTTG	
Exon 16F	AGCCTTTGAGCTTTGGTTTAG	369
Exon 16R	GATGCTGCCTCTGAGGAGAT	
Exon 17F	TTTACCGTTTCTTTCTGTTTCC	543
Exon 17R	CATTTCATTGTTGGGTGCTG	
Exon 18F	TAAAAGTGGGCATGAAAATCTTC	559
Exon 18R	TAGGTGGGTAATGTCAAGCAAT	

## Results

**Figure 1:**
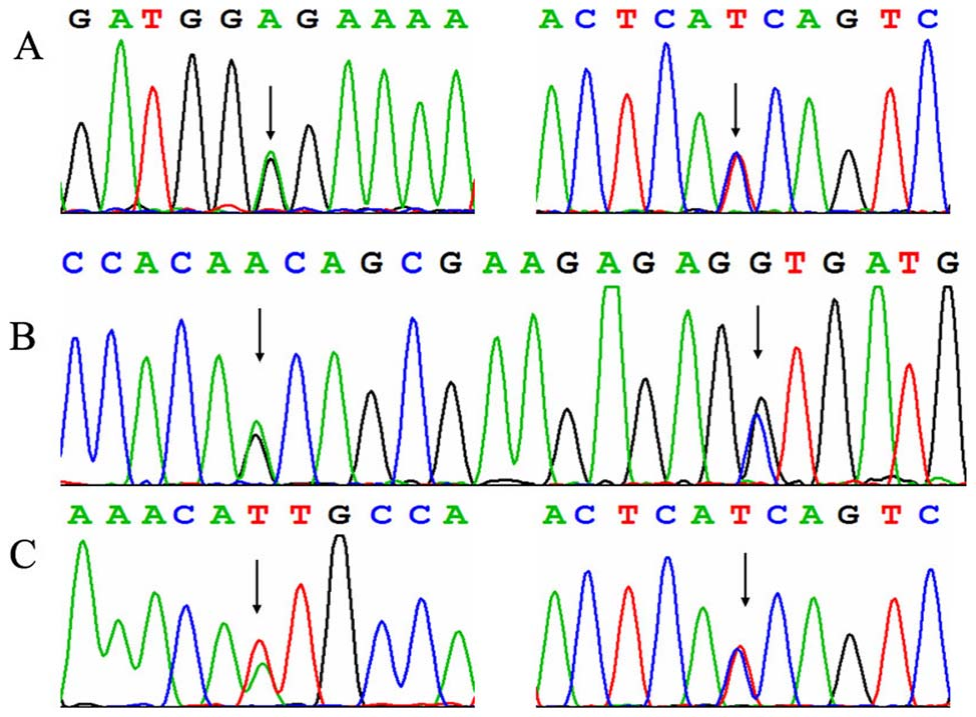
Electropherograms showing mutations in the *ALDH7A1* gene in 3 patients. A: c.410G>A (p.G137E), IVS11+1G>A (inverting sequencing) in case 1; B: heterozygous c.952 G>C (p.A318P), heterozygous c.965 C>T (p.A322V) in case 2; C: heterozygous c.902A>T (p.N301I), IVS11+1G>A (inverting sequencing) in case 3.

**Figure 2:**
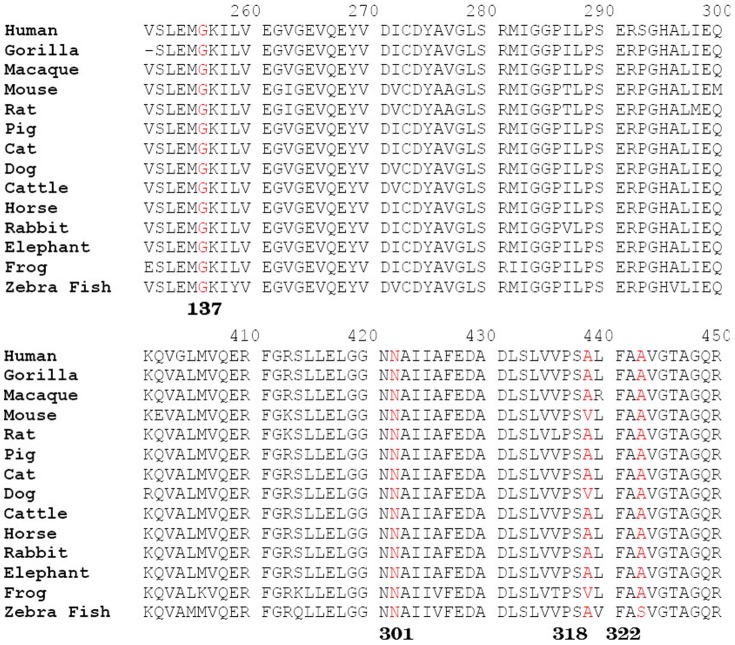
Evolutionary conservation of four novel *ALDH7A1* missense mutations identified in Chinese PDE patients.

*ALDH7A1* gene mutation analysis revealed compound heterozygous mutations respectively in the 3 patients (Figure 1). Five different mutations among the 6 alleles in *ALDH7A1* were identified in these patients. Both their parents had been available for testing and proved the autosomal recessive inheritance of the novel alleles. Case 1: heterozygous c.410G>A (p.G137E) in exon5 that was inherited from the father, and IVS11+1G>A in intron 11 inherited from the mother. Case 2: heterozygous c.952 G>C (p.A318P) in exon 11 that was inherited from the mother, and heterozygous c.965 C>T (p.A322V) in exon11 that was inherited from the father. Case 3: heterozygous c.902A>T (p.N301I) in exon10 that was inherited from the mother, and IVS11+1G>A in intron 11 inherited from the father. Four of these mutations were not reported before and the other one mutation (N301I) was reported, including missense and splice site mutations. The mutations G137E, A322V and N301I all occur in regions that are highly conserved in antiquitin across species, suggesting their evolutional importance (Figure 2). The 3 novel missense mutations, G137E, A318P and A322V were not detected in 50 unrelated Chinese controls. The novel splice site mutation IVS11+1 G>A occurred 2 times in Case 1 and Case 3. This mutation was predicted to result in the abolition of splice sites with the splice prediction tool (http://fruitfly.org/seq_tools/splice.html).

## Discussion

PDE is a rare autosomal recessive disorder. Before the confirmation of PDE by biochemical and/or genetic methods, difficult clinical diagnosis resulted in a prevalence that varied from 1∶20.000 to 1∶600.000 infants with epileptic encephalopathy in the UK [14]–[16]. As far as we know, this is the first time to report Chinese PDE patients diagnosed by mutation analysis. At the present time, the methods to examine the molecular markers of alpha-aminoadipic semialdehyde and pipecolic acid in body fluids have not been established in China, and blood or DNA samples are not easy to be sent outside of China. PDE is a clinical diagnosis. And good history and response to pyridoxine treatmentis diagnostic. Patients should be kept on pyridoxine. However, in China, at present doctor's diagnosis consciousness of PDE is lacking and the compliance of patients and their families is not good. All the above conditions limit the PDE cases to be diagnosed. Although metabolite and genetic tests do not need for the treatment of PDE patients, the genetic confirmation could further make physicians and patients more firmly believe the diagnosis and continue with pyridoxine treatment.

Based on the clinical manifestations and the response to treatment, PDE was considered in case one according to previous clinical diagnosis criteria [17]–[19]. This patient experienced seizure recurrence 3 times after pyridoxine treatment interruption. The intervals from pyridoxine withdrawal to seizure recurrence were 13, 14, and 38 days, respectively; and these intervals are similar to what has been reported in the literature (between 1 and 51 days) [6], [20]. However, the initial diagnosis of PDE in patient 1 was uncertain due to the following conditions: low Apgar scores resulting in hypoxic ischemic encephalopathy, the initial response to phenobarbital that prevented early pyridoxine testing, and difficulty in observing the independent effect of pyridoxine due to a combination of different antiepileptic drugs. The above difficulties in diagnosing PDE have also been reported in previous studies [16], [21]. Patient 2 showed seizure onset in the neonatal period. A transient low dose pyridoxine treatment during the course of disease did not show obvious effect. After administration of multiple antiepileptic drugs being ineffective, high-dose pyridoxine therapy was taken into account and the significant treatment effect guided a diagnostic PDE. Seizure onset age in patient 3 was earliest in our 3 patients. He experienced a similar tortuous course of treatment as patient 1, which was inadvertently given pyridoxine therapy with good results but not to attract attention of doctors. The treatment process of twists and turns in our 3 patients gave us a profound lesson and enhanced our identification of PDE from a clinical view.

Although several researchers reported EEG characteristics of PDE patients, no specific pattern of EEG abnormalities has been documented [22]–[25]. Abnormal background activity together with a variety of paroxysmal features has been described in most PDE patients. However, it is common to have normal interictal EEGs or only minimal epileptiform activity in some untreated patients, as well as in many pyridoxine-treated patients [26]. In our 3 patients, normal EEGs or only occasional multifocal discharges were repeatedly observed before pyridoxine therapy, suggesting that a clinical diagnosis of PDE should not be based solely on the effects of pyridoxine on the interictal EEG. The demonstration of the clinical efficacy of pyridoxine treatment is required to make a definitive diagnosis, as well as biochemical and/or genetic confirmation. Cranial magnetic resonance imaging shows a spectrum of changes from normal to hypoplasia of the corpus callosum, megacisterna magna [27], enlarged ventricles and diffusive cerebral hemispheric gray and white matter atrophy [6], [28]. Normal and nonspecific abnormal brain MRI were all observed in our patients.

The identification of the *ALDH7A1* gene mutations confirmed the diagnosis of PDE in our patients. The mutations of c.410G>A (p.G137E), c.952 G>C (p.A318P), c.965 C>T (p.A322V) and IVS11+1G>A (found in 2 patients) have not yet been reported. The first 3 mutations are all missense mutations, whereas the IVS11+1G>A mutation results from erroneous splicing. The mutation of c.902A>T (p.N301I) (NM_001182.4) or named heterozygous c.818A>T (p.N273I) (NM_001201377) is a known mutation [9]. Mills found that exons 4, 6, 9, 11 and 14 appear to be mutation ‘hot spots’, harbor 60% of reported mutations in Caucasian patients and are targets for an initial screen in Caucasian patients [6]. In our 3 Chinese patients, 3 of 5 different mutations among the 6 alleles in *ALDH7A1* were located in exon or intron 11. These data suggest that an initial screen of ALDH7A1 in Chinese patients should include the genes in these regions. To of the best of our knowledge, only 3 patients from Korea [11] and 2 patients from Japan [29] have been reported within 20 years in Asia. The diagnosis of Chinese PDE patients will be good for Chinese physicians further aware of PDE as treatable disease and the differential diagnosis of neonatal and infantile onset intractable epilepsy. In the future, more cases should be accumulated to improve the clinical diagnosis of this disease, and provide the *ALDH7A1* gene mutation spectrum in Chinese patients.

PDE patients need lifelong supplemental pyridoxine. There is no clear dose recommendation at present for long-term treatment. In most infants with PDE, therapeutic pyridoxine dosages vary from 15 to 30 mg/kg/day [8]. In our patients, the current dose was 120 mg/day. We do not plan to increase the dose of pyridoxine because seizures are well-controlled and intelligence is being improved. Whether to adjust the dose in the future depends on the condition of seizure control.

Because of the increase in alpha-aminoadipic semialdehyde, piperideine-6-carboxylate or pyridoxal phosphate cannot be corrected by pyridoxine therapy. PDE prognosis shows large differences in individual patients and the outcome of pyridoxine treatment is also variable. Even though normal intellectual function has been reported, most patients still have developmental delay and intellectual disability [9], [30], [31]. It is not known why some patients continue having seizures on high dose pyridoxine and have severe phenotype, while others have no seizures and close to normal development. Therefore, the neurodevelopmental prognosis of PDE might be associated with the unknown relationship between the *ALDH7A1* genotype and phenotype [20], [30]–[33]. In our patients, the definite diagnosis and correct treatment led to seizures free accompanied with improved neurodevelopment, but also below normal levels. The long-term prognosis will require further follow-up.
